# Association between rs20456 and rs6930913 of Kinesin-Like Family 6 and Hypertension in a Chinese Cohort

**DOI:** 10.1155/2021/1061800

**Published:** 2021-12-18

**Authors:** Yan-li Chen, Li-Qiang Zheng, Tie-Jun Li, Zhao-Qing Sun, Ying Hao, Bao-Gang Wu, Ying-Xian Sun

**Affiliations:** ^1^Department of Cardiology, The First Hospital of China Medical University, Shenyang, Liaoning, China; ^2^Department of Clinical Epidemiology, Library, Shengjing Hospital of China Medical University, Shenyang, Liaoning, China; ^3^Department of Cardiology, Shengjing Hospital of China Medical University, Shenyang, Liaoning, China; ^4^Department of Geriatrics, Taizhou First People's Hospital, Huangyan Hospital of Wenzhou Medical University, Taizhou, Zhejiang, China; ^5^Department of Geriatrics, Shengjing Hospital of China Medical University, Shenyang, Liaoning, China

## Abstract

This study aimed to investigate the relationship between kinesin-like family 6 (KIF6) polymorphisms and hypertension in a northeast Chinese cohort. In this study, two single nucleotide polymorphisms of KIF6 (rs20456 and rs6930913) and their haplotype were analyzed in 382 hypertension patients and 378 controls with SHEsis analysis platform, and the gene-environmental interactions were evaluated with logistic regression analysis. After adjusting for confounding factors, significantly lower risk of hypertension was observed in participants with genotype TC (0.416 (CI 0.299–0.578), *p* < 0.001) and CC (0.577 (0.389–0.857), *p*=0.007) of rs20456 compared with TT. For rs6930913, allele T (0.522 (0.386–0.704), *p* < 0.001), genotype TT (0.325 (0.205–0.515), *p* < 0.001), and genotype CT (0.513 (0.379–0.693), *p* < 0.001) were significantly associated with lower risk of hypertension than allele C and CC genotype, respectively. Gene-environment analyses confirmed the significant influence on hypertension by the interactions between genotypes distribution in rs20456 (CT: *p*=0.036, TT: *p*=0.022) and smoking status. No interactions were found between smoking and rs6930913, except those with dominant or recessive genetic models (both *P*_*s*_=0.006). There were no interactions between KIF6 and overweight (all *P*_*s*_ > 0.05). Haplotype analyses showed that CC (*p*=0.005) and TC (*p*=0.001) of rs20456 and rs6930913 were significantly associated with a statistically increased risk of hypertension. The false-positive report probability (FPRP) analysis was used to verify significant findings. In conclusions, KIF6 might affect the susceptibility of hypertension. The allele C (rs20456) and allele T (rs690913) were inclined to protect individuals from hypertension both in genotype and haplotype analyses.

## 1. Introduction

Hypertension is a major risk factor for cardiovascular and cerebrovascular diseases, such as coronary heart disease (CHD), stroke, heart failure (HF), and chronic kidney disease. Essential hypertension (EH), accounting for 90–95% of the total hypertension cases [1], is a polygenic disease. Genetic alterations, environmental factors, and gene-environmental interactions are supposed to have a key role in the etiology of EH [2].

Previously, we carried out a SNP discovery by Affymetrix Genome-Wide Human SNP Array 6.0 with pooled genomic DNA samples from 740 hypertensive patients and 361 normal controls (unpublished data), which revealed a significant association between kinesin-like family 6 (KIF6) and hypertension. KIF6 is a member of kinesins superfamily involved in the intracellular transport of protein complexes, membrane organelles, and messenger ribonucleic acid along microtubules [3]. KIF6 protein is ubiquitously expressed in the coronary arteries and vascular cells [4]. Many case-control studies have shown that the KIF6 gene is associated with CHD's risk [5, 6]. However, it remains unknown whether KIF6 is associated with hypertension or not. In this study, two tag SNPs (rs20456 (c.2180 + 130T > C) and rs6930913 (c.1427–798C > T)) of KIF6 with minor allele frequency ≥0.1 were selected to investigate the relationships between KIF6 and hypertension in a northeast Chinese Han cohort.

## 2. Materials and Methods

### 2.1. Study Population

A cross-sectional survey of 29,970 participants who were ≥35 years old was conducted by well-trained local doctors from 2004 to 2006 in the rural areas of Fuxin city in China. The survey and blood pressure (BP) measuring methods have been previously described in detail [7, 8]. Demographic data, including smoking and drinking status, body mass index (BMI), and antihypertensive medications, were recorded. Smoking status was defined as smoking cigarettes ≥1 piece a day for at least one year; overweight status was defined as BMI ≥25 kg/m^2^; hypertension was defined as average systolic blood pressure (SBP) ≥ 140 mmHg and/or an average diastolic blood pressure (DBP) ≥ 90 mmHg and/or self-reported current treatment for hypertension with antihypertensive medication [9].

The inclusion criteria for the hypertensive group were as follows: (1) age ≥45 years and ≤75 years, and (2) SBP ≥160 mmHg and/or DBP ≥90 mmHg or a history of stage 2 hypertension [9]. The inclusion criteria for control participants were as follows: (1) SBP <140 mmHg and DBP <90 mmHg, (2) no history or family history of hypertension, and (3) as old as possible. Individual with any of the following, secondary hypertension, pregnancy, severe renal or liver dysfunction, and cancer, was excluded. In qualified cases, 382 hypertensive patients and 378 normotensive controls were selected for further genotyping.

The study was approved by the Ethics Committee of China Medical University. Written informed consent was obtained from each participant.

### 2.2. Biochemical Test and DNA Extraction

A series of tests including a standard lipid panel, fasting blood sugar (FBS), serum ions (potassium, sodium, and chlorine), liver, and kidney assays were conducted with separated serum of peripheral blood in the Clinical Laboratory of General Hospital of the Fuxin Mining Bureau. Genomic DNA was extracted with TIANamp Blood DNA kits (Tiangen Biochemical Technology, Beijing, China) from EDTA anticoagulated peripheral blood and stored at −80°C until use. The concentration and OD260/280 of DNA, measured by Nanodrop 2000 (Thermo, American), were used to ensure the quality of DNA.

### 2.3. Genotyping of rs20456 and rs6930913

The SNPs of KIF6 for the Han Chinese population in Beijing, China (CHB), were downloaded from the International Genome Sample Resource (https://www.internationalgenome.org/). Two SNPs (rs20456 (c.2180 + 130T > C) and rs6930913 (c.1427–798C > T)) were selected by Haploview 4.2 (https://www.broadinstitute.org/haploview/haploview), with the criteria of *r*^2^ ≥ 0.8 and minor allele frequency (MAF) ≥ 0.1. The primers were designed by Primer Premier 5.0 (Premier, Canada). The polymerase chain reaction (PCR)-high resolution melting (HRM) curve analyses with LightCycler 480 (Roche) was applied to genotype SNPs in 2010. Detailed procedures of PCR and HRM are given in Table 1.

### 2.4. Statistical Analysis

The *t*-test or chi-square test was applied to assess continuous variables or categorical variables of baseline characteristics, respectively. The distribution of genotypes was tested for the Hardy–Weinberg equilibrium (HWE) with a chi-square test. Logistic regression analysis was used to explore the relationships between SNPs genotypes and hypertension and the influences of smoking or overweight status. Linkage disequilibrium (D′ and *r*^2^) between rs20456 and rs6930913 and haplotype frequencies were computed on the SHEsis statistical analysis platform. Odds ratios (OR) and 95% confidence intervals (CI) were calculated for all genotypes under different genetic models. The false-positive report probability (FPRP) was used to evaluate noteworthy associations of significant observations with the method described [10–12]. A prior probability of 0.1 was adopted to detect an OR of 1.5 for protective effects, and a threshold of 0.2 was considered as a noteworthy FPRP value. Statistical software package SPSS 21.0 (SPSS Inc., Chicago, IL, USA) and SHEsis platform (http://analysis.bio-x.cn/myanalysis.php) were used for statistical analyses.

## 3. Results

### 3.1. Baseline Characteristics

The baseline data for all participants are given in Table 2. The fraction of male participants and those of mean age were found significantly higher in the hypertensive group (both *P*_*s*_ < 0.001). The cases had a significantly higher BP and a higher serum level of low-density lipoprotein cholesterol (LDL–C), creatinine (Cr), sodium (Na^+^), as well as a higher BMI (all *P*_*s*_ < 0.001). There were no significant differences between cases and controls for smoking habit, FBS, triglyceride, or potassium levels (all *P*_*s*_ > 0.05). After adjusting for gender and age (<65 y and ≥65 y), smoking habit became significantly different in two groups (*p* < 0.001).

### 3.2. The Relationship between SNPs in KIF6 and Hypertension

Distributions of genotypes for rs20456 and rs6930913 were in equilibrium by HWE analysis (Table 3). Genotype distributions of rs20456 and rs6930913 in cases and controls and their associations with hypertension are given in Table 3. Gender, age (<65 y or ≥ 65 y), BMI (overweight or not), LDL-C, and Cr (<88.4 mmol/l or ≥ 88.4 mmol/l) were considered as adjusting factors. Compared with genotype TT, CC, or TC in rs20456 was statistically related with lower susceptibility to hypertension (all *P*_*s*_ < 0.05) after adjusting for variates. In the dominant genetic model, the risk of hypertension was statistically reduced too (*p* < 0.001). Allele T of rs6930913 significantly decreased the risk of hypertension, compared to the wild allele C (*p* < 0.001). Genotypes CT and TT of rs6930913 were significantly associated with a lower risk of hypertension, compared with genotype CC (both *P*_*s*_ < 0.01). Whether in the dominant or recessive genetic model of rs6930913, susceptibility to hypertension was significantly reduced (both *P*_*s*_ < 0.01). After adjusting for confounding factors, the differences still existed for rs6930913.

### 3.3. Gene-Environmental Interactions

The interactions of SNPs and smoking or overweight status on the risk of hypertension revealed that smoking significantly increased the susceptibility to hypertension for participants with rs20456 TT (*p*=0.022) after adjusting for covariates (Table 4). For rs6930913, significantly higher susceptibility to hypertension was found for smokers under the dominant or recessive genetic model (both *P*_*s*_=0.006) of rs6930913_T (probably protective). There were no interactions between overweight status and rs20456 or rs6930913 in relation to the risk of hypertension.

### 3.4. Linkage Disequilibrium Test and Haplotype Analysis

The linkage disequilibrium coefficients (D′ and *r*^2^) were calculated for rs20456 and rs6930913. The D′ and *r*^2^ were 0.76 and 0.34, respectively, which indicated a tight linkage between the two SNPs. Haplotypes of rs20456 and rs6930913 were constructed based on the linkage of the two SNPs (Table 5). The results showed that CT (*p* < 0.001) and TT (*p*=0.012) of rs20456 and rs6930913 were significantly associated with a lower risk of hypertension. Meanwhile, CC (*p*=0.003) and TC (*p*=0.001) of rs20456 and rs6930913 had reverse function on hypertension.

### 3.5. FPRP and Power Analysis

The FPRP was used to investigate the false-positive probability of preceding significant associations (*p* < 0.05) detected in this present study. The FPRP values for these remarkable results at different levels of prior probability and statistical power are given in Table 6. The results of the FPRP analysis confirmed the noteworthy associations of KIF6 rs20456 and rs6930913 polymorphism and hypertension susceptibility at the prior probability level of 0.25 (FPRP < 0.200). At the prior probability of 0.1, all the statistically significant findings were noteworthy except for the comparison of different alleles of rs20456 (C vs. T).

## 4. Discussion

This study explored significant relationships of two SNPs (rs20456 and rs6930913) in KIF6 with hypertension in a northeast Chinese Han cohort. The mean age of control participants was significantly higher than that of hypertensives due to the inclusion criteria. Because of gender difference in cardiovascular diseases distribution [13, 14], higher proportion of female was found for the control group in the present study. After adjusting for demographic baseline data (including gender and age), significantly lower risk of hypertension was observed in patients with genotype TC or CC of rs20456. Allele T and genotype CT or TT of rs6930913 were also significantly associated with a lower risk of hypertension after adjustment. Haplotypes CT and TT in rs20456 and rs6930913 were strongly associated with decreased risk of hypertension. The relationship between SNPs (rs20456 and rs6930913) of KIF6 and hypertension was modified by smoking status under some genetic models. In addition, the FPRP test suggested a truly significant relationship between KIF6 rs20456 and rs6930913 polymorphisms and hypertension susceptibility in the northern Chinese Han population.

The exact mechanisms of KIF6 in the cardiovascular system remain unclear. KIF6 gene (NM_145027, locus-6p21.2) spans over 23 exons and encodes kinesin, which mediates the intracellular transport of organelles, protein complexes, and messenger ribonucleic acids [15]. KIF6 protein has been detected in a variety of tissues, including coronary arteries and other vascular tissues [16]. KIF6 polymorphisms have been reported to be associated with epicardial coronary endothelial dysfunction, interrupting intracellular transport in endothelial cells to develop CHD and different statin treatment outcomes [5, 17–22]. According to a recent study, KIF6 has a specific role for endothelial cells ciliogenesis in vertebrates [23]. Endothelial dysfunction is also closely related to hypertension and atherosclerotic coronary and cerebral artery disease [24–27]. Improvement of endothelial function was reported to reduce blood pressure in spontaneously hypertensive rats [28, 29]. Studies of sex differences on endothelial function are conflicting [30]. Age-related endothelial dysfunction [31–33], artery stiffness [34, 35], and low-grade inflammation [36] have been reported close relationships with cardiovascular diseases. It is unknown whether the age and gender-related differences of endothelial dysfunction relate to kinesins. Different age, gender, and blood pressure might also be the various phenotypes coming from the same genetic tree of KIF6 and other genes. This study investigated close relationship between KIF6 and hypertension after adjusting for demographic characteristics, which might provide one genetic mechanism for hypertension.

Hypertension is a multifactorial genetic disorder modified by environmental and epigenetic factors [37]. This study's gene-environmental analysis showed that the associations between SNPs (rs20456 and rs6930913) in KIF6 and hypertension could be partly modified by smoking. As a result, for patients with smoke susceptivity genotypes, stopping smoking may contribute to their health. Interactions between kinesin and smoking need to be further investigated.

Hypertension has been the most common condition in developed countries, and its costs and social burden are increasing rapidly [38, 39]. The etiology of hypertension is complicated. It is important to know the genetic bases and gene-environmental interactions for prevention and treatment of hypertension. As far as we know, this is the first report on the relationship between KIF6 and hypertension. There are several limitations to this study. First, more SNPs or other genetic marks should be analyzed to ensure the relationship between KIF6 and hypertension. Second, a larger number of samples are needed to investigate the relationship. Finally, future studies should also include other races due to genetic heterogeneity among different human species. In all, additional investigations are warranted to elucidate the pathophysiological functions of KIF6 in hypertension.

## 5. Conclusion

The present study has identified a close relationship between KIF6 SNPs (rs6930913 and rs20456) and hypertension, modified by smoking status. Considering the genotype and haplotype analyses, the allele C in rs20456 and allele T of rs690913 were inclined to protect individuals from hypertension.

## Figures and Tables

**Table 1:** PCR profiles (primers, amplicon size, and Tm) of rs20456 and rs6930913 in KIF6.

SNP	Primers (5′ to 3′)	Amplicon size (bp)	Tm (°C)
rs20456	F: CCTGTGGCTCTCATGTCTCTTG	113	56
	R: GCACTCCAACCAACTTGAAAGC		

rs6930913	F: TTCATTCTGTGTTCACGATGC	150	58
	R: GAGCCTTCTCTAGCGATGC		

SNP, single nucleotide polymorphism.

**Table 2:** General characteristics of all individuals.

Characteristics	Cases (*n* = 382)	Controls (*n* = 378)	*P* value	*P* ^*∗*^ value
Gender (male/female)	220/162	168/210	**0.001**	
Age (years)	58.67 ± 8.62	62.43 ± 4.88	**<0.0001**	
SBP (mmHg)	179.69 ± 19.21	120.54 ± 10.42	**<0.0001**	
DBP (mmHg)	108.74 ± 9.00	74.85 ± 6.81	**<0.0001**	
Smoking (%)	184 (48.2%)	206 (54.5%)	0.082	**<0.0001**
Having	184	206		
Never	198	172		
BMI (kg/m^2^)	25.08 ± 3.44	22.60 ± 3.55	**<0.0001**	**<0.0001**
LDL-C (mmol/l)	3.31 ± 2.12	2.85 ± 0.74	**<0.0001**	**<0.0001**
TG (mmol/l)	1.64 ± 1.66	1.67 ± 1.14	0.809	
Cr (*µ*mol/l)	86.72 ± 13.68	52.30 ± 11.19	**<0.0001**	**<0.0001**
FBG (mmol/l)	5.63 ± 1.77	5.73 ± 1.59	0.432	
Na^+^ (mmol/l)	143.54 ± 2.22	142.45 ± 2.42	**<0.0001**	**<0.0001**
K^+^ (mmol/l)	4.27 ± 0.51	4.23 ± 0.35	0.254	

BMI, body mass index; Cr, serum creatinine; DBP, diastolic blood pressure; FBG, fasting plasma glucose; K^+^, potassium; LDL-C, low-density lipoprotein cholesterol; Na^+^, sodium; SBP, systolic blood pressure; TG, triglyceride; smoking (having), current or past smoking; smoking (never), never smoked. Statistically significant differences (*p* < 0.05) are marked in bold. ^*∗*^Adjusting for gender and age.

**Table 3:** Association of KIF6 gene polymorphisms with hypertension susceptibility.

rs no.	Allele or genotype	Cases (*n* = 382)	Controls (*n* = 378)	Or (95% CI)	*P* value	Or (95% CI)^*∗*^	*P* value^*∗*^
rs20456	T	410 (53.7%)	382 (50.5%)	Ref		Ref	

HWE = 0.96	C	354 (46.3%)	374 (49.5%)	0.939 (0.850–1.039)	0.221	0.731 (0.552–0.967)	0.028
	TT	118 (30.9%)	88 (23.3%)	Ref		Ref	
	TC	174 (45.5%)	206 (54.5%)	**0.630 (0.495–0.802)**	**<0.0001**	**0.416 (0.299–0.578)**	**<0.0001**
	CC	90 (23.6%)	84 (22.2%)	0.799 (0.600–1.065)	0.125	**0.577 (0.389–0.857)**	**0.007**
	Dominant (CC + CT vs. TT)	**0.679 (0.540–0.853)**	**0.001**	**0.460 (0.338–0.625)**	**<0.0001**		
	Recessive (CC vs. CT + TT)	**1.473 (1.173–1.850)**	**0.001**	1.009 (0.721–1.412)	0.960		
rs6930913	C	538 (70.4%)	420 (55.5%)	Ref		Ref	

HWE = 0.34	T	226 (29.6%)	336 (44.5%)	**0.733 (0.664–0.809)**	**<0.0001**	**0.522 (0.386–0.704)**	**<0.0001**
	CC	184 (48.6%)	124 (32.8%)	Ref		Ref	
	CT	170 (44.9%)	172 (45.5%)	**0.666 (0.488–0.909)**	**0.010**	**0.513 (0.379–0.693)**	**<0.0001**
	TT	28 (6.5%)	82 (21.7%)	**0.230 (0.142–0.374)**	**<0.0001**	**0.325 (0.205–0.515)**	**<0.0001**
	Dominant (TT + CT vs. CC)	**0.525 (0.392–0.705)**	**<0.0001**	**0.460 (0.346–0.610)**	**<0.0001**		
	Recessive (TT vs. CT + CC)	**0.286 (0.181–0.450)**	**<0.0001**	**0.454 (0.293–0.704)**	**<0.0001**		

^*∗*^Adjusting for gender, age (<65 year,  ≥ 65 year), body mass index (normal, overweight), serum level of low-density lipoprotein cholesterol, and creatinine (<88.4 mmol/l, ≥ 88.4 mmol/l). Statistically significant differences (*p* < 0.025) are marked in bold. SNP, single nucleotide polymorphism; HWE, Hardy–Weinberg equilibrium.

**Table 4:** Gene-environmental interactions on the risk of hypertension.

	Smoking		BMI (kg/m^2^)	
	Ever/never	*P* ^*∗*^	≥25/<25	*P* ^*∗*^
rs20456				
CC	—	—	—	—
TC	11.941 (1.181–120.777)	**0.036**	0.160 (0.017–1.537)	0.112
TT	20.234 (1.537–266.334)	**0.022**	0.081 (0.006–1.152)	0.064
Dominant (TT + CT/CC)	6.895 (1.014–46.862)	0.048	0.151 (0.021–1.075)	0.059
Recessive (TT/CT + CC)	3.773 (0.592–24.032)	0.160	0.493 (0.081–2.998)	0.443

rs6930913				
CC	—	—	—	—
CT	0.112 (0.012–1.014)	0.051	0.812 (0.112–5.878)	0.836
TT	10.303 (0.854–124.331)	0.066	7.846 (0.690–89.207)	0.097
Dominant (TT + CT/CC)	16.290 (2.193–120.980)	**0.006**	1.367 (0.235–7.946)	0.728
Recessive (TT/CT + CC)	16.500 (2.218–122.725)	**0.006**	6.608 (0.987–44.234)	0.052

^*∗*^Adjusting for gender, age, low-density lipoprotein cholesterol, and creatinine. BMI, body mass index. Statistically significant differences (*p* < 0.025) are marked in bold.

**Table 5:** Haplotypes of KIF6 gene in cases and controls.

Haplotype rs20456-rs6930913	Cases	Controls	Or (95% CI)	*P*
CC	242 (31.7%)	188 (24.9%)	1.402 (1.120–1.756)	**0.003**
CT	112 (14.7%)	186 (24.6%)	0.535 (0.405–0.682)	**<0.0001**
TC	296 (38.7%)	232 (30.7%)	1.449 (1.154–1.764)	**0.001**
TT	114 (14.9%)	150 (19.8%)	0.695 (0.543–0.927)	**0.012**

Statistically significant differences (*p* < 0.025) are marked in bold.

**Table 6:** Results of false-positive report probability analysis for the risk associations of rs20456 and rs6930913 polymorphisms to hypertension.

Genotype and variables	Or (95% CI)	*P* value^a^	Statistical power^b^	Prior probability				
				0.25	0.1	0.01	0.001	0.0001
rs20456 T > C								
C vs. T	0.731 (0.552–0.967)	0.028	0.741	**0.102** ^*∗*^	0.255	0.790	0.974	1.000
TC vs. TT	0.416 (0.299–0.578)	<0.0001	0.002	**0.000** ^*∗*^	**0.001** ^*∗*^	**0.007** ^*∗*^	**0.065** ^*∗*^	0.411
CC vs. TT	0.577 (0.389–0.857)	0.006	0.237	**0.075** ^*∗*^	**0.196** ^*∗*^	0.729	0.964	0.996
CC/CT vs. TT	0.460 (0.338–0.625)	<0.0001	0.009	**0.000** ^*∗*^	**0.001** ^*∗*^	**0.008** ^*∗*^	**0.072** ^*∗*^	0.437
rs6930913 C > T								
T vs. C	0.522 (0.386–0.704)	<0.0001	0.054	**0.001** ^*∗*^	**0.003** ^*∗*^	**0.036** ^*∗*^	0.273	0.790
CT vs. CC	0.513 (0.379–0.693)	<0.0001	0.044	**0.001** ^*∗*^	**0.003** ^*∗*^	**0.030** ^*∗*^	0.237	0.756
TT vs. CC	0.325 (0.205–0.515)	<0.0001	0.001	**0.005** ^*∗*^	**0.014** ^*∗*^	**0.132** ^*∗*^	0.606	0.939
TT/CT vs. CC	0.460 (0.346–0.610)	<0.0001	0.005	**<0.0001** ^*∗*^	**<0.0001** ^*∗*^	**0.001** ^*∗*^	**0.014** ^*∗*^	**0.122** ^*∗*^
TT vs. CT/CC	0.454 (0.293–0.704)	0.001	0.043	**0.028** ^*∗*^	**0.080** ^*∗*^	0.491	0.907	0.990
Haplotype rs20456–rs6930913								
CC	1.402 (1.120–1.756)	0.003	0.722	**0.013** ^*∗*^	**0.039** ^*∗*^	0.309	0.819	0.978
CT	0.535 (0.405–0.682)	<0.0001	0.038	**<0.0001** ^*∗*^	**<0.0001** ^*∗*^	**0.001** ^*∗*^	**0.012** ^*∗*^	**0.105** ^*∗*^
TC	1.449 (1.154–1.764)	0.001	0.635	**0.001** ^*∗*^	**0.003** ^*∗*^	**0.033** ^*∗*^	0.257	0.776
TT	0.695 (0.543–0.927)	0.013	0.611	**0.061** ^*∗*^	**0.164** ^*∗*^	0.683	0.956	0.995

CI, confidence interval; OR, odds ratio. ^a^Chi-square test was used to calculate the genotype frequency distributions. ^b^Statistical power was calculated using the number of observations in each subgroup and the corresponding ORs and *p* values in this table. ^*∗*^The level of false-positive report probability threshold was set at 0.2, and noteworthy findings are marked in bold.

## Data Availability

The data used to support the findings of this study are available from the corresponding author upon request.

## References

[1] Patnaik M., Pati P., Swain S. N. (2014). Aldosterone synthase C-344T, angiotensin II type 1 receptor A1166C and 11-*β* hydroxysteroid dehydrogenase G534A gene polymorphisms and essential hypertension in the population of Odisha, India. *Journal of Genetics*.

[2] Briggs F. B. S., Hill E., Abboud H. (2021). The prevalence of hypertension in multiple sclerosis based on 37 million electronic health records from the United States. *European Journal of Neurology*.

[3] Mundy D. I., Machleidt T., Ying Y. S., Anderson R. G., Bloom G. S. (2002). Dual control of caveolar membrane traffic by microtubules and the actin cytoskeleton. *Journal of Cell Science*.

[4] Miki H., Okada Y., Hirokawa N. (2005). Analysis of the kinesin superfamily: insights into structure and function. *Trends in Cell Biology*.

[5] Hamidizadeh L., Haji Hosseini Baghdad Abadi R., Babaee Baigi M. A., Dastsooz H., Khazaei Nejhad A., Fardaei M. (2015). Impact of KIF6 polymorphism rs20455 on coronary heart disease risk and effectiveness of statin therapy in 100 patients from southern Iran. *Archives of Iranian Medicine*.

[6] Ruiz-Ramos D., Hernández-Díaz Y., Tovilla-Zárate C. A. (2015). The Trp719Arg polymorphism of the KIF6 gene and coronary heart disease risk: systematic review and meta-analysis. *Hereditas*.

[7] Chen Y.-L., Li T.-J., Hao Y. (2018). Association of rs2271037 and rs3749585 polymorphisms in CORIN with susceptibility to hypertension in a Chinese Han population: a case-control study. *Gene*.

[8] Sun Z., Zheng L., Wei Y. (2007). Prevalence and risk factors of the rural adult people prehypertension status in Liaoning Province of China. *Circulation Journal*.

[9] Chobanian A. V., Bakris G. L., Black H. R. (2003). Seventh report of the joint national committee on prevention, detection, evaluation, and treatment of high blood pressure. *Hypertension*.

[10] Wacholder S., Chanock S., Garcia-Closas M., El Ghormli L., Rothman N. (2004). Assessing the probability that a positive report is false: an approach for molecular epidemiology studies. *Journal of the National Cancer Institute*.

[11] He J., Wang M. Y., Qiu L. X. (2013). Genetic variations of mTORC1 genes and risk of gastric cancer in an Eastern Chinese population. *Molecular Carcinogenesis*.

[12] He J., Zou Y., Liu X. (2018). Association of common genetic variants in pre-microRNAs and neuroblastoma susceptibility: a two-center study in Chinese children. *Molecular Therapy - Nucleic Acids*.

[13] Benjamin E. J., Muntner P., Alonso A. (2019). Heart disease and stroke statistics-2019 update: a report from the American Heart Association. *Circulation*.

[14] Whelton P. K., Carey R. M., Aronow W. S. (2018). ACC/AHA/AAPA/ABC/ACPM/AGS/APhA/ASH/ASPC/NMA/PCNA guideline for the prevention, detection, evaluation, and management of high blood pressure in adults: a report of the American college of cardiology/American heart association task force on clinical practice guidelines. *Hypertension*.

[15] Povel C. M., Boer J. M., Onland-Moret N., Dollé M. E., Feskens E. J., Van Der Schouw Y. T. (2012). Single nucleotide polymorphisms (SNPs) involved in insulin resistance, weight regulation, lipid metabolism and inflammation in relation to metabolic syndrome: an epidemiological study. *Cardiovascular Diabetology*.

[16] Hameed A., Bennett E., Ciani B. (2013). No evidence for cardiac dysfunction in Kif6 mutant mice. *PLoS One*.

[17] Aulchenko Y. S., Ripatti S., Lindqvist I. (2009). Loci influencing lipid levels and coronary heart disease risk in 16 European population cohorts. *Nature Genetics*.

[18] Ruiz-Iruela C., Padró-Miquel A., Pintó-Sala X. (2018). KIF6 gene as a pharmacogenetic marker for lipid-lowering effect in statin treatment. *PLoS One*.

[19] Vaccarino V., Badimon L., Corti R. (2011). Ischaemic heart disease in women: are there sex differences in pathophysiology and risk factors?: position Paper from the Working Group on Coronary Pathophysiology and Microcirculation of the European Society of Cardiology. *Cardiovascular Research*.

[20] Vishnuprabu D., Geetha S., Bhaskar L. V. K. S., Mahapatra N. R., Munirajan A. K. (2015). Genotyping and meta-analysis of KIF6 Trp719Arg polymorphism in south Indian coronary artery disease patients: a case-control study. *Meta Gene*.

[21] Wu G., Li G.-B., Dai B. (2012). Association of KIF6 variant with lipid level and angiographic coronary artery disease events risk in the Han Chinese population. *Molecules*.

[22] Yoshino S., Cilluffo R., Prasad M. (2016). Sex-specific genetic variants are associated with coronary endothelial dysfunction. *Journal of the American Heart Association*.

[23] Konjikusic M. J., Yeetong P., Boswell C. W. (2018). Mutations in Kinesin family member 6 reveal specific role in ependymal cell ciliogenesis and human neurological development. *PLoS Genetics*.

[24] Berenyiova A., Drobna M., Cebova M., Kristek F., Cacanyiova S. (2018). Changes in the vasoactive effects of nitric oxide, hydrogen sulfide and the structure of the rat thoracic aorta: the role of age and essential hypertension. *Journal of Physiology and Pharmacology : An Official Journal of the Polish Physiological Society*.

[25] Kelleher R. J., Soiza R. L. (2013). Evidence of endothelial dysfunction in the development of Alzheimer’s disease: is Alzheimer’s a vascular disorder?. *American Journal of Cardiovascular Disorder*.

[26] Panza J. A., Casino P. R., Kilcoyne C. M., Quyyumi A. A. (1993). Role of endothelium-derived nitric oxide in the abnormal endothelium-dependent vascular relaxation of patients with essential hypertension. *Circulation*.

[27] Ras R. T., Streppel M. T., Draijer R., Zock P. L. (2013). Flow-mediated dilation and cardiovascular risk prediction: a systematic review with meta-analysis. *International Journal of Cardiology*.

[28] Azis N. A., Agarwal R., Ismail N. M. (2019). Blood pressure lowering effect of Ficus deltoidea var kunstleri in spontaneously hypertensive rats: possible involvement of renin-angiotensin-aldosterone system, endothelial function and anti-oxidant system. *Molecular Biology Reports*.

[29] Fu J., Han Y., Wang J. (2016). Irisin lowers blood pressure by improvement of endothelial dysfunction via AMPK-akt-eNOS-NO pathway in the spontaneously hypertensive rat. *Journal of the American Heart Association*.

[30] Stanhewicz A. E., Wenner M. M., Stachenfeld N. S. (2018). Sex differences in endothelial function important to vascular health and overall cardiovascular disease risk across the lifespan. *American Journal of Physiology - Heart and Circulatory Physiology*.

[31] Ungvari Z., Labinskyy N., Gupte S., Chander P. N., Edwards J. G., Csiszar A. (2018). Dysregulation of mitochondrial biogenesis in vascular endothelial and smooth muscle cells of aged rats. *American Journal of Physiology-Heart and Circulatory Physiology*.

[32] Thum T., Hoeber S., Froese S. (2007). Age-Dependent impairment of endothelial progenitor cells is corrected by growth hormone mediated increase of insulin-like growth factor-1. *Circulation Research*.

[33] Pierce G. L., Lesniewski L. A., Lawson B. R., Beske S. D., Seals D. R. (2009). Nuclear factor-*κ*b activation contributes to vascular endothelial dysfunction via oxidative stress in overweight/obese middle-aged and older humans. *Circulation*.

[34] Bretón-Romero R., Wang N., Palmisano J. (2016). Cross-sectional associations of flow reversal, vascular function, and arterial stiffness in the Framingham Heart Study. *Arteriosclerosis, Thrombosis, and Vascular Biology*.

[35] Kohn J. C., Lampi M. C., Reinhart-King C. A. (2015). Age-related vascular stiffening: causes and consequences. *Frontiers in Genetics*.

[36] Sanada F., Taniyama Y., Muratsu J. (2018). Source of chronic inflammation in aging. *Frontiers in Cardiovascular Medicine*.

[37] Lin J., Lin S., Wu Y., Wang X., Wu S., Li H. (2017). Hypomethylation of the angiotensin II type I receptor (AGTR1) gene along with environmental factors increases the risk for essential hypertension. *Cardiology*.

[38] Wierzejska E., Giernaś B., Lipiak A., Karasiewicz M., Cofta M., Staszewski R. (2020). A global perspective on the costs of hypertension: a systematic review. *Archives of Medical Science*.

[39] Bielecka-Dabrowa A., Aronow W. S., Rysz J., Banach M. (2011). The rise and fall of hypertension: lessons learned from Eastern Europe. *Current Cardiovascular Risk Reports*.

